# The Potential Genetic Effect for Yield and Foliar Disease Resistance in Faba Bean (*Vicia faba* L.) Assessed via Morphological and SCoT Markers

**DOI:** 10.3390/plants12203645

**Published:** 2023-10-22

**Authors:** Alaa A. Soliman, Manar I. Mousa, Abeer M. Mosalam, Zeinab E. Ghareeb, Shafik D. Ibrahim, Medhat Rehan, Haitian Yu, Yuhua He

**Affiliations:** 1 Food Legumes Research Department, Field Crops Research Institute, Agricultural Research Center, Giza 12619, Egypt; manaribrahimmousa@gmail.com; 2 Institute of Food Crops, Yunnan Academy of Agricultural Sciences, Kunming 650205, China; haitianlegume@outlook.com; 3 Department of Agronomy, Faculty of Agriculture, Damietta University, New Damietta 34511, Egypt; abeer.mosalam@du.edu.eg; 4 Center Laboratory for Design and Statistical Analysis Research, Agricultural Research Center, Giza 12619, Egypt; zeinabghareeb@yahoo.com; 5 Agricultural Genetic Engineering Research Institute, Agricultural Research Center, Giza 12619, Egypt; shafikdarwish@ageri.sci.eg; 6 Department of Plant Production and Protection, College of Agriculture and Veterinary Medicine, Qassim University, Buraydah 51452, Saudi Arabia; m.rehan@qu.edu.sa; 7 Department of Genetics, Faculty of Agriculture, Kafrelsheikh University, Kafr El-Sheikh 33516, Egypt

**Keywords:** genetic variability, heterosis, heritability, genetic advance, *Vicia faba*, molecular markers, SCoT, foliar diseases

## Abstract

Faba bean is considered one of the most prominent grain legumes, with high protein content for human food consumption and livestock feed. The present study evaluated the nature of gene action and determined the genetic diversity among different populations of three crosses for resistance to foliar diseases at the molecular level. Analysis of variance exposed significant differences among the generations for all measured traits. Both dominance and additive gene effects were essential, but dominance genes, for the most part, exhibited greater effects than additive ones. This indicates an essential role for dominant genes alongside the additives one in inheriting such traits. The third cross (Marina × Giza 40) gave desired significant and positive (additive × additive) values for the number of pods/plant, seeds/plant, and seed yield/plant, in addition to desirable negative values for chocolate spot and rust characteristics. Furthermore, assessing the lines under study using seven SCoT primers disclosed three bands with recorded molecular weights of 260, 207, and 178 bp, generated by SCoT-1, SCoT-4, and SCoT-7 primers, respectively. These bands exist in the resistant parent (Marina), which could be attributed to the high-disease-resistance phenotypes, and they are absent in the sensitive parent (Giza 40) and other putative sensitive lines. Based on the molecular profiles and the genetic similarity between parents and the selected lines, the highest similarity value (0.91) was detected between Marina genotype and BC_1_, revealing a high foliar disease resistance. Meanwhile, Giza 40 (susceptible to foliar diseases) exhibited the maximum value (0.93) with F_2_. Additionally, cluster analysis based on genetic relationships was performed, and a high level of correlation between the results of PCR-based SCoT analysis and the foliar disease reactions was observed in the field. Consequently, this study concluded that SCoT markers created reliable banding profiles for evaluating genetic polymorphism among faba bean lines, which could be a foundation for developing an efficient breeding program.

## 1. Introduction

Faba bean (*Vicia faba* L.) is one of the most prominent cool-season grain legumes cultivated worldwide as a vital source of protein for human food and animal feed uses. Faba bean plays a role in contributing to nitrogen fixation and soil biodiversity in cereals cropping systems [1,2]. It is a diploid species with 2n = 12 that belongs to the Fabaceae family and can be divided into three subspecies based on seed size including large-seeded type (*V. faba subsp major*), medium-seeded type (*V. faba subsp *equina**), and small-seeded type (*V. faba subsp minor*). The cross-pollination in faba beans has been observed to range from 8% to 84%, with an average of around 30% to 60% depending on the climatic factors and genotypes [3,4,5].

The Faba bean are classified as the fourth crop among legume crops after pea, chickpea, and lentil and are considered the seventh most produced legume [6,7], with fully mature (dry seeds) production reaching 4.84 million tons in 2017 [8]. In Egypt, its productivity recorded 170,236 metric tons of total production from the total harvested area of 120,116 faddan (1 faddan = 0.42 ha) with a seed yield average of 1.42 tons/faddan (Economic Affairs Sector, Ministry of Agriculture 2021). However, the yield is still low due to the partial adaptability of available cultivars to wide-ranging environments and disease susceptibility [9,10].

Foliar and soil-borne diseases are major limiting factors in legume production since chocolate spot and rust are the major diseases affecting Faba bean productivity and quality. Two different pathogens cause chocolate spot diseases. *Botrytis fabae* sard is the most severe and the more specific disease in Egypt, especially in humid areas. Moreover, this disease has been reported to cause a dense reduction in yields, reaching over 60% of yield by destroying the plant leaves and limiting photosynthetic activity [11,12,13,14]. The second disease is the Faba rust caused by *Uromyces viciae-fabae* (Pers.), a widespread disease with the highest identified virulence rate in the Egyptian populations. The virulence tendency varies from moderate to severe, with yield losses ranging 22–26% from the yield [15,16,17].

In faba bean germplasm, only a few sources of genetic resistance to chocolate spots have been known [18,19]. Different tactics may apply for controlling chocolate spot, and more effort is needed in order to find resistant materials adapted to different environments [10,20]. Furthermore, improving resistance genotypes for foliar diseases is the most vital objective for a breeding program. Breeders use several biometrical methods to formulate the most efficient breeding systems for estimating the genetic effects of genes controlling quantitative traits to improve the breeding program. Thus, generation means analysis, gene action, inbreeding depression, genetic parameters, and anticipated genetic gain from character selection provide the essential evidence for plant breeders to predict the effective breeding program. These parameters can be used in the first generations to obtain maximum improvement and to enhance yield potentials amongst a large selection [21,22].

The six-parameter model analysis includes parents (P_1_ and P_2_), first and second generations (F_1_ and F_2_), and the first two backcrosses (BC_1_ and BC_2_) is considered the best analysis method. This method was suggested by Gamble [23] and supplies breeders with the early genetic information of selected genotypes.

Heterosis and inbreeding depression are essential for explaining the genetic parameters in faba bean. Heterosis for seed yield is estimated to indicate heterotic effects in yield components. Consequently, heterosis resulting from the combined action and interaction between allelic and inter-allelic genes is an effective means by which faba bean and hybrid combinations will improve yield [22,24]. The heterotic effects in faba bean ranged from significantly positive to significantly negative estimates, contributing to the improvement in yield and its components [25,26]. Inbreeding depression reduces auto fertility and yield in the absence of pollinator, and yield can be minimized by 11% through the loss of heterosis, whereas high inbreeding depression recorded lower values in the F_2_s, in general, than the F_1_s for most traits in faba bean [26,27,28].

Estimating heritability is essential for predicting the expected genetic advance through selection in segregating populations. Genetic advances help us understand gene action’s nature, which expresses many polygenic characteristics. High values of genetic advance indicate additive gene action, whereas low values point to non-additive gene action; subsequently, the heritability estimation will be dependable if escorted by a high genetic advance [29].

The success of any breeding program depends on the presence of sufficient genetic variability among used genotypes to permit effective selection. Mean analysis was developed to estimate the variance of genetic components and the acceptable knowledge obtained about the mode of inheritance and the nature of gene effects. The gene effects are essential for developing high-yielding varieties in faba bean [12]. The yield potential remains unrealized in faba bean mainly due to the lack of success in hybrid breeding for the exploitation of heterosis. Therefore, improvements in seed yield and yield stability addressed through the component traits, namely, resistance against different pathogens and pests, are considered major breeding objectives in faba bean enhancement [25].

Assessing genetic variation is crucial in plant breeding as it enables the selection of genotypes that exhibit favorable traits [30]. Various molecular markers have been used to demonstrate plant and other organisms’ genetic variation [31,32]. Molecular markers are fast, reliable, repeatable, unaffected by environmental conditions, and used to demonstrate genetic variation and select important plant agricultural characteristics [33]. Many molecular techniques, such as random amplification of polymorphic DNAs (RAPDs), restriction fragment length polymorphisms (RFLPs), target region amplification polymorphisms (TRAPs), sequence-specific amplification polymorphisms (SSAPs), and amplified fragment length polymorphisms (AFLPs), were implied to detect the genetic variability of *Vicia* species and *V. faba* L. populations [30]. The start codon targeted (SCoT) marker system is a relatively new molecular tool used in plant research that targets conserved regions flanking the ATG start codon in plant genes [34]. This technique has many advantages, including the fact that it does not require prior sequence information, allowing it to be used with a wide range of plant species; it is highly reproducible and cost-effective; and it is linked to functional genes or regions surrounding these genes and their corresponding traits [35,36,37]. Remarkably, based on the SCoT marker simplicity (it only requires a single primer for amplification), it produces a high percentage of polymorphism and an abundance of available genetic information [38].

The present study aimed at (i) determining the behavior of gene action and heritability of yield and its components in three crosses of faba bean using six populations; (ii) exploring the inbred depression in yield and its components in the same crosses and populations; (iii) assessing the genetic variation among selected lines at the molecular level; (iv) developing some molecular genetic markers (SCoT) associated with resistance and susceptibility to foliar diseases. The previous measurements are essential for improving foliar disease tolerance in faba bean varieties.

## 2. Results

### 2.1. The Performance of Crosses

The mean performance and variance of all the studied traits in the three crosses (Nubaria 5 × Sakha 1, Nubaria 5 × Giza 40 and Marina × Giza 40) for the six populations (P_1_, P_2_, F_1_, F_2_, BC_1_, and BC_2_) were presented in Table 1 Results revealed a difference between every two parents in all studied traits of the three crosses. The parental genotypes could be classified into two groups, the first group included the genotypes of Nubaria 5 (P_1_), Sakha 1 (P_2_), and Marina (P_4_), which are considered resistant to foliar diseases. On the contrary, the second group involved Giza 40 (P_3_), showed high susceptibility to foliar diseases, accompanied by affected yield components and seed yield/plant.

The highest number of pods/plant was observed in the first cross of P_2_, F_1_, BC_1_, and BC_2_, with recorded values of 42.27, 49.47, 40.20, and 46.42, respectively. Moreover, P_2_, BC_1_, and BC_2_ in the first cross had the best numbers of seeds/plant (142.40, 148.47, and 157.39), respectively, while F_1_ and F_2_ manifested the uppermost numbers in the second cross (168.60 and 126.47).

The mean values of P_2_, F_1_, BC_1_, and BC_2_ in the first cross verified the highest seed yield/plant (110.94, 121.14, 115.61, and 112.83 gm), respectively, whereas the third cross produced the minimum values of seed yield/plant. However, BC_1_ and BC_2_ displayed the plants least infected with chocolate spot and rust diseases, especially in the third cross. In general, F_1_ mean value revealed the tallest plants (149.00 cm) and the highest number of seeds/plant (168.60) in the second cross; meanwhile, it had the highest number of branches/plant (7.00), pods/plant (49.47), and seed yield/plant (121.14) in the first cross. 

The heterosis, inbreeding depression, and potency ratio of the three crosses for the studied traits are mentioned in Table 2. In most cases, all traits revealed significant and/or highly significant and positive heterotic effects regarding heterosis over better and mid-parent (BP and MP). Results showed positive and significant (*p* ≤ 0.05 and *p* ≤ 0.01) heterosis over mid-parent in all the measured traits through the three crosses except a chocolate spot in the second and third crosses and a rust trait in the first cross, which showed a significant and negative effect. Meanwhile, heterosis over better parent recorded the same trend except for some traits such as plant height in the third cross, chocolate spot in the first and second crosses, and rust in the first cross. In this light of that, both types of heterosis disclosed the desired best values of positive heterotic effects regarding the number of seeds/plant, number of branches/plant, seed yield/plant, and pods/plant in all crosses. However, the desired negative values of heterotic effects were detected with chocolate spot and rust traits in the second and first crosses, respectively. 

Applying the inbreeding depression revealed positive values for all traits except for a chocolate spot in the first and second crosses and rust diseases in the first and third crosses. Inbreeding depression significantly or highly significantly (*p* ≤ 0.05 and *p* ≤ 0.01) and positively increased and produced the maximum number of branches/plant in the first and third crosses, the number of pods/plant in the first cross, and chocolate spot and rust in the third and second crosses. Regarding the potence ratio, values greater than unity (*p* > +1) reflect over-dominance towards the better parent for most cases. Over-dominance manifested for all evaluated traits under different crosses except for the chocolate spot in all crosses, plant height, number of pods/plant, and rust trait in the third cross with scored values less than unity, demonstrating a partial dominance effect. 

Applying scaling tests, A, B, C, and D (Table S1) revealed significant and positive or negative differences for all the assessed traits through the three crosses, indicating the presence of non-allelic interactions and the inadequacy of the simple model in interpreting the differences between population means. Hence, generation means as genetic analysis produces genetic parameters such as (m) mean effect, (a) additive, (d) dominance, (aa) additive × additive, (ad) additive × dominance, and (dd) dominance × dominance, as previously conducted by Gamble [23]. These assessed parameters in the three crosses are mentioned in Table 3. The mean effect (m) showed highly significant (*p* ≤ 0.01) differences in the three crosses for all the evaluated characteristics, which specified the inheritance of quantitative effects. The effects of the additive gene (a) presented a highly significant effect (*p* ≤ 0.01) in the positive or negative direction for plant height in all three crosses, plus number of branches/plant, number of pods/plant, and rust through the first cross, in addition to the rust trait in the second cross. The estimates of the dominance gene effect (d) manifested a positive and highly significant effect (*p* ≤ 0.01) for plant height and number of branches/plant in the first cross, whereas the first and second crosses exhibited the same behavior with respect to number of pods/plant, number of seeds/plant, and seed yield/plant.

Meanwhile, dominance with a negatively significant effect (*p* ≤ 0.01) was expressed for chocolate spot and rust traits (negative values are eligible) in the second and third crosses.

When going forward and estimating the values of additive × additive (aa), a positively significant effect for plant height, number of branches/plant, and seed yield/plant in the first cross was detected, whereas traits such as number of pods/plant and number of seeds/plant recorded this positive effect in both first and third crosses. Meanwhile, a values were negative for plant height, number of pods/plant, and number of seeds/plant in the second cross, as well as for foliar diseases in the second and third crosses. Doing the same analysis for additive × dominance (ad) demonstrated a highly significant and positive behavior through all the assessed traits in the third cross except two traits (number of pods/plant and rust), whereas the second cross disclosed the same trend with positive effect in traits such as plant height and foliar diseases. When applying dominance × dominance (dd) assessment, the second cross exhibited a desirable and highly significant effect regarding traits such as plant height, number of pods/plant, and number of seeds/plant.

The three crosses assessed for all measured traits in relation to heritability in broad (h^2^_b_) and narrow (h^2^_n_) senses, in addition to genetic advance (GS), are presented in Table 4. The heritability, in a broad sense, recorded the best values in the third cross for number of seeds/plant (90.1), whereas the first cross recorded the uppermost value (92.2, 91.63) for foliar diseases (chocolate spot and rust), respectively. Meanwhile, the lowest value was recorded (57.28) for seed yield/plant in the second cross. The estimated value of heritability in a narrow sense using the data of F_2_ and backcross were evaluated, and they presented from high to moderate values for most traits. Traits including number of pods/plant and number of seeds/plant in the second cross, in addition to chocolate spot and rust in the first cross increasing to the maximum values. On the other hand, the lowest values were assigned for number of branches in the third cross and seed yield/plant in the second cross. 

Regarding the genetic advance under selection (GS%), the number of pods/plant and the number of seeds/plant in the first cross had the best percentages (80.3 and 77.55), respectively. Meanwhile, the minimum values were detected in plant height trait in all crosses.

### 2.2. Polymorphism via SCoT Markers

**Figure 1:**
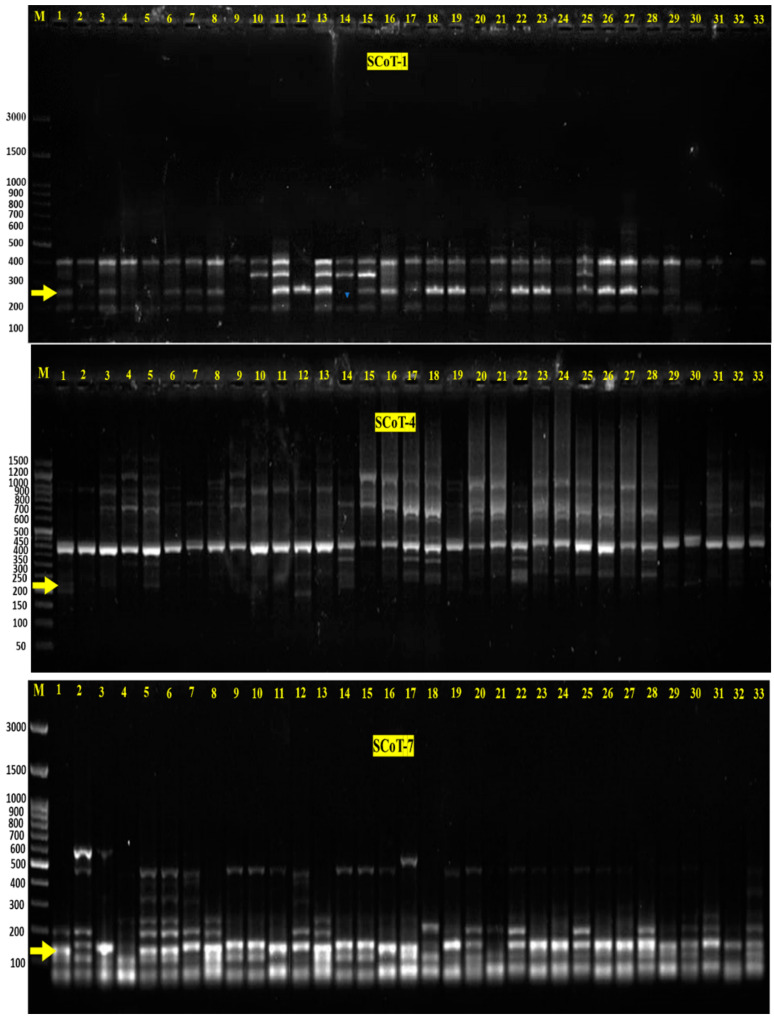
SCoT-PCR amplification using primers SCoT1, SCoT4, and SCoT7, respectively. The yellow arrows point to the generated bands 260, 207, and 178 bp by primers SCoT1, SCoT4, and SCoT7, respectively.

SCoT-PCR analysis revealed that 522 total reliable bands were scored among the studied lines (Figure 1 and Figure S1 and Table 5). The maximum number of bands were produced by the SCoT-7 primer (116 bands), while the minimum number of bands were produced by the SCoT-3 primer (47 bands). A total of 165 bands were monomorphic, with an average of 24 bands per primer, ranging from 0 bands (SCoT-3, SCoT-4, SCoT-5, and SCoT-6) to 66 bands (SCoT-1 and SCoT-2). Furthermore, about 357 bands were polymorphic, ranging from 11 bands (SCoT-2) to 83 (SCoT-7), with an average of 51 bands per primer. The percentage of polymorphism ranged from 14% (SCoT-2) to 100% (SCoT3, SCoT4, SCoT5, and SCoT6), with an average of 74% per primer. Parents-specific bands that could be related to foliar disease are mentioned in Table 5. SCoT1, SCoT4, and SCoT7 patterned three positive bands in the resistant parent (Marina) that were absent in the sensitive parent (Giza 40), including the following: (1) A 260 bp (SCoT-1) is present in most of the resistant lines, while being absent in 32 and 33, which are considered sensitive lines in addition to 9, 10, 21, 30 and 31, which show moderate foliar disease resistance; (2) A 207 bp (SCoT-4) was present in lines 3, 5, 14, 29, and 30; and (3) A 178 bp (SCoT-7) was presented in the lines 5, 6, 8, 28, 29, and 30. Considering the previous results, the preceding lines showed foliar disease resistance, except 9, 10, 21, 29, 30, and 31, which reveal moderate foliar disease resistance.

On the other hand, five negative bands were observed with the sensitive parent (Giza 40), and these bands were absent in the resistant parent (Marina). The absence segments involved the following: 327 bp (SCoT-2) in 12, 13, and 30; 361 bp (SCoT-3) in 12,13, 22, and 23; 270 bp (SCoT-5) in F2 9, 10, 11, 12, 13, 30, and 31; 779 bp (SCoT-5) in 13, 19, and 29; 392 bp (SCoT-6) in 11, 12, 13, 19, 20, 22, 23, 32, and 33. Taking this into consideration, all the evaluated lines showed foliar disease susceptibility, except 9, 10, 11, 19, 20, 30, and 31, which exhibited moderate foliar disease resistance.

### 2.3. Genetic Parameters

Genetic parameters are critical for the effectiveness of a polymorphism-based marker technique used in discriminatory genotypes. Table 6 shows that several genetic parameters were estimated using the online Marker Efficiency Calculator (iMEC) to evaluate the informative and discriminatory power of subsequent faba bean generations. Heterozygosity represents the direct count of heterozygosity in the population and is estimated based on the allele frequency of individuals in the population according to the Hardy–Weinberg equilibrium. The heterozygosity index (H) was obtained and ranged from 0.416 (primer SCoT-1) to 0.491 (primer SCoT-6), with an average of 0.462. Moreover, the polymorphism index content (PIC) analysis was performed to determine the efficiency of each SCoT primer in expressing polymorphic loci in lines under evaluation. The calculated (PIC) values for primers ranged from 0.330 (SCoT-1 and SCoT-7) to 0.371 (SCoT-6), with an average of 0.355. Going forward, the effective multiplex ratio (E) was assessed and pointed to 1.424 with primer SCoT-3 to 3.515 with SCoT-7 primer, with an average of 2.260. The arithmetic means heterozygosity (H.av) ranged from 0.0024 for SCoT-5 primer to 0.0050 for SCoT-6 primer, with an average of 0.0034. The highest value of the marker index (MI) was detected with SCoT-1 and SCoT-7 primer (0.0089), while the minimum value was 0.0049 and revealed by SCoT-3 primer with an average of 0.0074. The discriminating power (D) of the applied primers ranged from 0.505 (SCoT-1) to 0.875 (SCoT-3), with an average of 0.701. Resolving power (RP) is used to describe the capacity of the marker combination to detect the differences among various lines. RP values of the primers varied from 0.485 (SCoT-1) to 3.152 (SCoT-5), with an average of 1.818. 

### 2.4. Genetic Distance and Similarity

Table S3 summarizes the estimated genetic distance between lines according to the Dice similarity coefficient. The genetic distance reflects the genetic relationships and the direction of the genetic improvement process. The data showed that the genetic similarity between pairs of lines ranged from 0.42 to 0.96. The biggest genetic similarity value was observed between BC_1_ (1) and both BC_1_ (2) and BC_1_ (3); these lines shared a high resistance to foliar diseases, whereas the lowest value of genetic similarity was detected among F_2_ (2) and F_2_ (6), with a contradictory ability towards resistance to foliar diseases. Concerning Marina’s parents, genetic similarity values ranged from 0.63 to 0.91, since the uppermost genetic similarity value was found between Marina and BC_1_ (5) with high foliar disease resistance.

In comparison, the lowest genetic similarity value manifested among Marina and BC_2_ (7). The previously mentioned lines exhibited a contradictory ability toward foliar disease resistance. Concerning Giza 40 parent, genetic similarity values ranged from 0.60 to 0.93. Giza 40 and F_2_ (10) manifested the highest genetic similarity value while exhibiting susceptibility to foliar diseases. Contrarily, the minimum value in genetic similarity was among Giza 40 and F_2_ (6), with a contradictory ability towards foliar disease resistance.

### 2.5. Phylogeny Analysis of ScoT Markers

**Figure 2:**
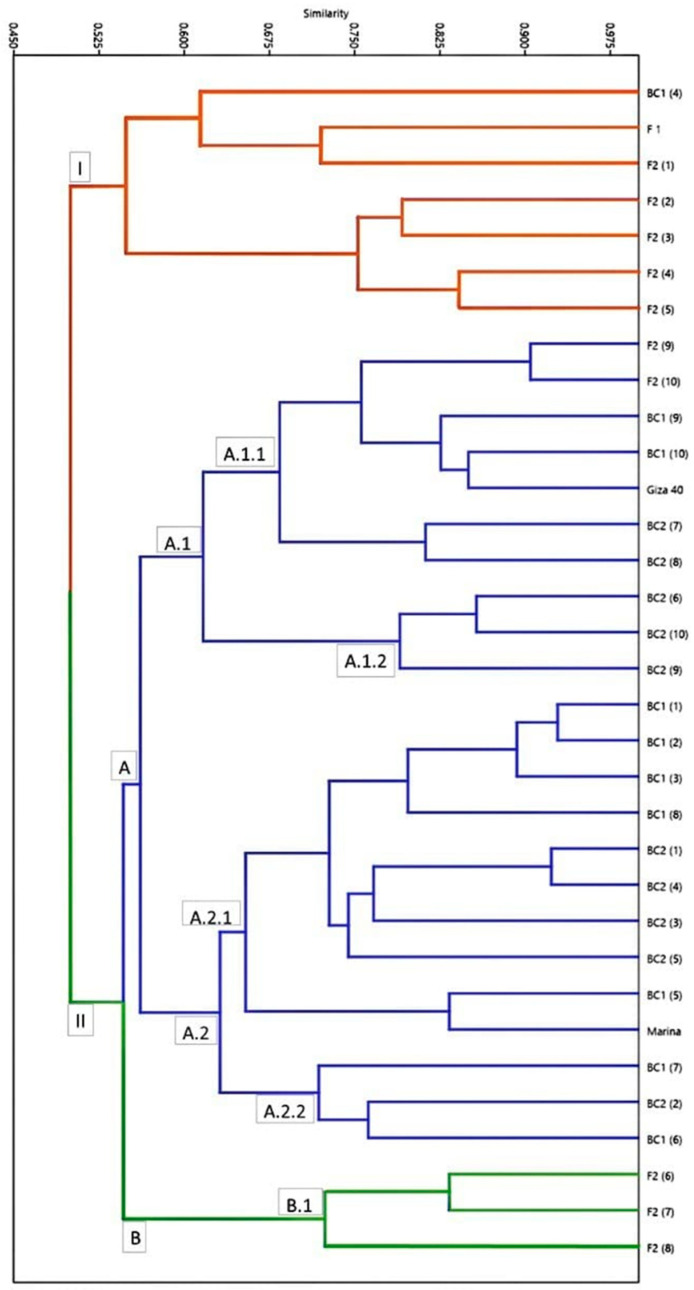
Dendrogram for the 33 selected lines constructed from a similarity matrix computed according to the Dice coefficient.

Cluster analysis derived from SCoT markers using UPGMA is presented in Figure 2. The selected 33 lines were grouped into two main clusters. Cluster I included seven lines (F_1_, F_2_ (1), F_2_ (2), F_2_ (3), F_2_ (4), F_2_ (5), and BC_1_ (4)), which showed resistant patterns for foliar diseases. Additionally, cluster II consisted of twenty-six lines further divided into sub-clusters: sub-cluster II.A and sub-cluster II.B. Sub-cluster II.A. involved 2 sub- sub-clusters (sub-sub-cluster II.A.1 had 10 lines divided into two groups (II.A.1.1 and II.A.1.2)). First, the II.A.1.1 group comprised seven lines (five of them, namely, Giza 40, F_2_ (9), F_2_ (10), BC_1_ (9), and BC_1_ (10), participated in the susceptibility to foliar diseases, whereas the other two lines, namely, BC_2_ (7) and BC_2_ (8), shared moderate resistant to foliar diseases). Second, group II.A.1.2 contained three lines, namely, BC_2_ (6), with moderate resistance, in addition to BC_2_ (9) and BC_2_ (10), which have susceptibility to foliar diseases. Furthermore, sub- sub-cluster II.A.2 involved 13 lines divided into two groups: the first group (II.A.2.1) had 10 lines, namely, Marina, BC_1_ (1), BC_1_ (2), BC_1_ (3), BC_1_ (5), BC_1_ (8), BC_2_ (1), BC_2_ (3), BC_2_ (4), and BC_2_ (5), since all of them exhibited a resistant reaction to foliar diseases except BC_1_ (8, which showed moderate resistance). Finally, sub-cluster II.B involved two sub- sub-clusters (II.B.1 and II.B.2), including three lines, namely, F_2_ (6), F_2_ (7), and F_2_ (8), which shared moderate resistance to foliar diseases. 

## 3. Discussion

Faba bean (*Vicia faba* L.) is considered one of the most popular and important legume crops in the Mediterranean and Central Asia (its native area of origin). It can be used as a protein source in human food consumption in addition to livestock feed based on its high protein content in seed and straw. Biotic stresses such as foliar diseases and *Orobanche* affect faba bean production and quality since early disease development during flowering or the season could cause large losses and severe damage [39,40]. 

Concerning chocolate spot and rust diseases, BC_1_ and BC_2_ revealed the lowest numbers of infected plants, especially in the third cross, and this may refer to to the first parent (Marina P_1,_) in these crosses, which was not adapted for the other environmental factors. Likewise, this genotype (Marina) is resistant to foliar diseases, mainly to chocolate spot, especially in F_1_, F_2_, BC_1,_ and BC_2_ plants, in addition to rust in BC_1_ and BC_2_ when compared with the other two crosses. 

It was observed that F_1_ mean values were higher than other populations and mostly exceeded the high parent with respect to most evaluated traits in the three crosses, which may refer to over-dominance. Then, selection could effectively improve these traits in the next generation, particularly the number of seeds/plant and seed yield/plant. Results obtained in all measured traits established that the variance of the segregated generations (F_2_ and BCs) was greater than the non-segregated generations (P_1_, P_2_, and F_1_), pointing to the environmental effects on the gene expression and effectiveness of selection for these traits. Therefore, parents are precisely selected to find the desired recombination in the segregated generations, and these results are in consonance with the findings of prior published reports by Ashrei et al., Hendawy, Koumber and El-Gammaal, Akhshi et al. [22,41,42,43].

The heterosis percentage is a deviation of mean performance for the F_1_ average from the mid-parent or better parent with respect to the traits. Both types of heterosis exhibited the best values and positive heterotic effects in the number of seeds/plant, number of branches/plant, seed yield/plant, and pods/plant in all crosses, whereas the desired negative values of heterotic effects were observed in the second and first crosses with chocolate spot and rust traits, respectively. These results indicated that the dominance direction was toward the best respective parent, and the heterotic effect might be due to the dominance and/or dominance × dominance effects. These findings are in harmony with those obtained by Ashrei et al. [22]. Hybrid vigor means that trait analysis recorded statistically positive and significant different results. This means that the F_1_ superior to the mid-parent might reflect the genetic variability in crossed parents and vice versa [26,44]. The value of inbreeding depression is the opposite of the heterosis value. In the case of crossing between inbreed lines, their progeny may achieve higher performance than them (hybrid vigor), as reported by Falconer [45]. The reduction in the mean performance of F_2_ indicates an increment in inbreeding depression and vice versa, which implies the low genetic diversity of the parents [22,26,46]. In the potence ratio, values of measured characteristics, more than unity, reflect over-dominance effects. Most of the evaluated traits had an over-dominance effect, suggesting that the selection must be delayed to late generations [42].

In the current study, the dominance degree appeared higher than the additive gene effect for most traits in the three crosses, indicating the significance of additive and dominance gene effects playing a vital role in the inheritance of these traits. Moreover, we can select desirable characters in the early generations, but the last generations are influential.

The gene effect of (aa) was relatively more important than the additive effects, whereas the dominance effect was less important. Furthermore, the effects of (dd) are higher in magnitude than the epistatic gene effects (aa) for all traits under study in the three crosses except the number of branches and number of pods/plant in the first cross, and number of seeds/plant and seed yield/plant in the third cross. The previous findings suggest that selection plays a vital role if delayed till dominance, and its epistatic effects are reduced to the lowest value, slowing down the progress of selection. These results agreed with the research reported by Akhshi et al., Attia and Salem, and Salama and Mohamed [43,47,48,49].

Heritability estimation of different traits is essential for crop improvement programs and for predicting the response of selection depending on the magnitudes of genetic variance components for additive and dominance. High heritability, in a broad sense, indicates the essential role for the inheritance of additive- and non-additive-effect genes related to these traits. Moreover, high heritability in a narrow sense, indicates that the genetic variance of additives played a vital role in the existence of variability. These results are similar to those reported previously [22,26,44,46]. Johnson et al. [50] reported that to predict selection results for the best individuals, we need to estimate the heritability value with genetic advances, which are more valuable than the value of heritability alone. Otherwise, Dixit et al. [51] disclosed that a high value of heritability is not always associated with a high genetic advance value. In this manuscript, the high value of genetic advance was associated with high and moderate heritability, in a narrow sense, for number of pods/plant in the first and third crosses, number of seeds/plant in the first cross, and seed yield/plant in the first and third crosses. Notably, moderate and low heritability plus predicted genetic advance indicated that the selection of faba bean in subsequent generations would be relatively more effective than in the early generation of F_2_. Similar findings were depicted by Abd El-zaher, Ashrei et al., Obiadalla-Ali et al., Abou-Zaid, and Aziz and Osman [21,22,44,52,53].

The obtained results revealed a high level of polymorphism, reflecting high divergence in the studied faba bean lines; subsequently, the SCoT technique is efficient in differentiating the lines under study and can be used to evaluate genetic variation among closely related cultivars. Our findings are in consonance with those reported by Nosair [54], who produced one hundred and eighty-three bands (183), generating 93.99% polymorphism of various Leguminosae species via SCoT markers. Furthermore, Essa et al. [55] observed a high level of polymorphism (reaching 70.93%) using six SCoT-PCR primers screened across eight faba bean cultivars. The present results indicate that the resistance genes present in faba bean genotypes may differ. However, more genotyping is still required in order to validate the suitability of these markers for marker-assisted faba bean breeding, and these loci could be effectively used in breeding programs. Heiba et al. [56] demonstrated that molecular markers exhibited interest-specific loci relating to faba bean genotypes’ resistance to chocolate spot, whereas Bosily et al. [57] detected specific markers for barley leaf rust disease using five Scot primers.

The polymorphism index content (PIC) is performed to determine the efficiency of each SCoT primer in expressing polymorphic loci into the selected faba bean lines [58]. The PIC values are more than 0.5 for highly informative markers, between 0.25–0.5 for reasonably informative markers, and less than 0.25 for slightly informative markers [59]. The discrimination power of a marker represents its ability to distinguish between closely related genotypes; additionally, it can aid in the efficient evaluation of various markers [60]. These findings indicated good sources of diversity, which will aid breeders in assessing genetic diversity and the relationship between different genotypes. The results confirmed that SCoT markers created trustworthy banding profiles, which were able to evaluate the genetic polymorphism among selected lines. The obtained findings coincide with those of Albrifcany and Askander [61], who confirmed that one could rely on the SCoT technique to evaluate the genetic diversity among the faba bean cultivars. The assessment of genetic similarity and genetic distance among plant cultivars helps adjust breeding programs to facilitate the selection of desirable genotypes among segregating and/or backcrossing populations [62]. The results suggested that the SCoT markers showed considerable potential for identifying and discriminating faba bean lines via their resistance to foliar diseases. Their use for evaluating chocolate spot disease resistance in faba bean genotypes has been depicted by Heiba et al. [56]. The current results showed that the SCoT marker technique is reliable in dividing sorts based on genetic distance [63]. Finally, differences in lines clustering patterns may be due to marker sampling error, polymorphism level, or the number of loci and their coverage across the genome [64].

## 4. Materials and Methods

### 4.1. Field Experiment

This investigation was conducted at the Food Legumes Department, Sakha Agricultural Research Station, Agriculture Research Center (ARC), Egypt, during three growing winter seasons: 2019/2020, 2020/2021, and 2021/2022. Four genotypes of faba bean (Nubaria 5, Sakha 1, Giza 40 (belonging to *V. faba subsp equina*), and Marina (belonging to *V. faba subsp minor*)) were crossed in six population designs. The pedigree, origin, and reaction to foliar diseases of these genotypes are presented in Table 7.

In the 2019/2020 season, the four faba bean genotypes were crossed in six populations fashioned under insect-free cages to obtain the F_1_ hybrid seeds of three crosses as follows: (Nubaria 5 × Sakha 1), (Nubaria 5 × Giza 40), and (Marina × Giza 40). In the 2020/2021 sowing season, the F_1_ seeds and their parents were planted, and F_1_ plants were self-pollinated to obtain F_2_ and backcrossed to both parents to obtain BC_1_ and BC_2_ for each cross. Re-hybridization was performed on the three crosses to obtain enough F_1_ hybrid seeds under the same insect-free cage.

In the last season (2021/2022), a field experiment was constructed to evaluate the six populations’ seeds (P_1_, P_2_, F_1_, F_2_, BC_1_, and BC_2_) for the three crosses in a randomized complete block design (RCBD) in three replicates. Two ridges represented each entry for parents and F_1′_s, eight ridges for F_2′_s, and three ridges for each BC. The ridge was 3 m long and 60 cm apart. Seeds were planted on one side of the ridge at 20 cm hill spacing with one seed per hill. Data were recorded as an average of 15, 20, 100, and 30 on individual guarded plants from each plot for each cross’s parents (P1 and P2), F_1_, F_2_, and BC (BC_1_ and BC_2_) generations, respectively. All recommendations related to faba bean agronomy and production applied during the growing seasons. Data were recorded on plant height (cm), number of branches/plant, number of pods/plant, number of seeds/plant, seed yield/plant (g), and foliar diseases (chocolate spot and rust). The chocolate spot and rust scale was 1–9 (1: highly resistant and 9: highly susceptible). Foliar disease reactions were measured according to Bernier et al. [65].

### 4.2. Statistical Analysis

Analysis of variance and mean performance of the studied traits measured the differences among the evaluated generations [66]. The mean analysis among generations was performed using the method of Mather and Jinks [67] after the procedure scaling test to detect the presence of epistasis (non-allelic interaction).

### 4.3. Types of Gene Effect (Gene Action)

Data were analyzed to estimate the mean effects (m), additive (a), dominance (d), additive × additive (aa), additive × dominance (ad), and dominance × dominance (dd) via genetic analysis of the mean between generation according to Gamble [23].

Heterosis and inbreeding depression are calculated according to the method of Mather and Jinks [67], whereas the potency ratio was calculated based on the method of Petr and Frey [38] as follows:

$$\mathrm{PR}=F_{1}-\bar{MP}/0.5(\bar{HP}-\bar{LP}),$$

where: F_1_ = mean performance of F_1′_; MP = value of mid parent; HP = high parent value; LP = low parent value.

Heritability was estimated in broad (h^2^_b_) and narrow (h^2^_n_) senses as mentioned by Mather [68], whereas predicted genetic advance (GS) was calculated as previously stated by Miller et al. [69].

### 4.4. DNA Extraction and SCoT Amplification Marker

To identify some molecular genetic markers associated with resistance and susceptibility to foliar diseases, the total genomic DNA was extracted by the DNA easy Plant Mini Kit (QIAGEN, Hilden, Germany) from the 14-day leaves of 33 lines [the parent Marina (resistant, coded 1), Giza 40 (susceptible, coded 2), the promising F_1_ hybrid (Marina X Giza 40, coded 3), 10 selected lines from each of F_2_ (coded 4–8 resistant, 9–11 moderate, and 12–13 susceptible), BC1 (Marina X Giza 40 with Marina, coded 14–18 resistant, 19–21 moderate, and 22–23 susceptible), and BC2 (Marina X Giza 40 with Giza 40, coded 24–28 resistant, 29–31 moderate and 32–33 susceptible)). A NanoDrop spectrophotometer was used to estimate the concentration and quality of extracted DNA. Seven SCoT primers were implied to detect polymorphism, as shown in Table S2. The amplification reaction was carried out in a 20 μL reaction volume containing 10 μL Master Mix (sigma), 2 μL primer (10 pcmol), 2 μL template DNA (10 ng), and 6 μL dH_2_O, according to Ibrahim et al. [67]. PCR amplification was accomplished in a Perkin-Elmer/GeneAmp^®^ PCR System 9700 (PE Applied Biosystems) programmed to fulfill 40 cycles after an initial denaturation cycle for 5 min at 94 °C. Each cycle consisted of a denaturation step at 94 °C for 45 s, an annealing step at 50 °C for 50 s, and an elongation step at 72 °C for 1 min. The primer extension segment was extended to 7 min at 72 °C in the final cycle. The PCR products were resolved by electrophoresis in a 1.5% agarose gel containing ethidium bromide (0.5 μg/mL) in 1× TBE buffer at 95 volts. Gels containing bands were visualized and photographed under UV light using a Gel Documentation System (BIO-RAD 2000).

### 4.5. PCR Scoring and Data Analysis

For SCoT analysis, only clear and unambiguous bands were visually scored as either present (1) or absent (0) for all samples using Gel Analyzer software [70], and the final datasets included both polymorphic and monomorphic bands.

The Online Marker Efficiency Calculator (iMEC software) [71] was used to compute seven basic measures of polymorphism indices for individual markers, such as iMEC calculates heterozygosity index (H), polymorphism information content (PIC), discriminating power (D), effective multiplex ratio (E), marker index (MI), arithmetic mean heterozygosity (Hav), and resolving power (R).

Then, a binary statistic matrix was constructed and Dice’s similarity matrix coefficients were calculated between lines using the unweighted pair group method with arithmetic averages (UPGMA) [72]. Using the PAST software Version 1.91 [73], this matrix was used to create a phylogenetic tree (dendrogram) based on Jaccard similarity coefficients [74]. 

## 5. Conclusions

The present study used six populations (P_1_, P_2_, F_1_, F_2_, BC_1_, and BC_2_) of three faba bean crosses (Nubaria 5 × Sakha 1; Nubaria 5 × Giza 40; and Marina × Giza 40). The scaling test showed that all of the evaluated traits significantly differed, indicating non-allelic interactions. BC_1_ and BC_2_, in the first cross, verified the highest seed yield/plant, and they displayed the least infected plants in the third cross with respect to chocolate spot and rust diseases (negative values are desirable). Both heterosis types disclosed the desired positive heterotic effects regarding the number of seeds/plant, number of branches/plant, seed yield/plant, and pods/plant in all crosses, whereas the desired negative values of heterotic effects were detected with respect to chocolate spot and rust traits in the second and first crosses, respectively. Moreover, broad-sense heritability recorded the best values in the third cross for number of seeds/plant, whereas the first cross revealed the uppermost values for foliar diseases (chocolate spot and rust), respectively. The molecular profile for the cross Marina × Giza 40 and some selected lines of F_1_, F_2_, BC_1_, and BC_2_ against seven SCoT primers generated 522 loci (357 polymorphic bands and 165 monomorphic bands), with average polymorphism (%) values reaching 74%. Three bands of molecular weight 260, 207, and 178 bp, generated via SCoT-1, SCoT-4, and SCoT-7 primers, respectively, were distinguished as being associated with the resistant parent (Marina). Based on the molecular profiles, genetic similarity among selected lines was estimated and found to range between 0.24 ang 0.96. The highest value (0.91) observed between Marina and BC1 (5) revealed a high resistance to foliar disease. Consequently, the SCoT markers can distinguish between lines for resistance or sensitivity to foliar diseases in developing an efficient breeding program.

## Figures and Tables

**Table 1:** Mean performance and variance for the six populations of the three crosses for the studied traits.

Traits	Crosses	Mean and Variance	Parameters					
			P_1_	P_2_	F_1_	F_2_	BC_1_	BC_2_
Plant Height	Cross 1	Mean	107.33	105.67	121.00	120.33	134.33	129.03
		Variance	35.24	60.24	36.43	153.27	115.06	88.31
	Cross2	Mean	107.33	128.43	149.00	118.65	111.50	105.45
		Variance	35.24	20.53	29.29	128.70	91.64	99.06
	Crosse 3	Mean	141.67	128.43	140.33	122.70	132.00	112.83
		Variance	41.67	20.53	48.10	134.04	83.79	97.73
No. of Branches/plant	Cross 1	Mean	4.33	5.20	7.00	4.02	5.40	4.11
		Variance	0.95	0.74	1.14	2.89	1.63	2.50
	Cross 2	Mean	4.33	4.50	5.00	4.80	5.00	5.18
		Variance	0.95	0.96	1.29	3.86	3.24	2.03
	Cross 3	Mean	1.60	4.50	5.93	4.92	4.83	4.73
		Variance	0.54	0.96	0.21	3.29	1.87	3.24
No. of Pods/Plant	Cross 1	Mean	37.47	42.27	49.47	29.51	40.20	46.42
		Variance	64.12	39.21	39.70	241.36	56.65	247.34
	Cross 2	Mean	37.47	41.43	47.67	41.56	34.20	39.27
		Variance	64.12	50.24	56.10	265.01	131.82	194.31
	Cross 3	Mean	31.27	41.43	38.07	30.36	38.33	37.20
		Variance	26.78	50.24	38.07	274.49	164.57	204.51
No. of Seeds/Plant	Cross 1	Mean	112.27	142.40	158.27	107.88	148.47	157.39
		Variance	481.07	221.69	246.21	2860.71	1161.57	2387.56
	Cross 2	Mean	112.27	111.14	168.60	126.47	104.73	111.48
		Variance	481.07	214.69	445.69	2588.01	1324.20	1724.36
	Cross 3	Mean	67.87	111.14	123.13	95.68	113.43	96.23
		Variance	97.98	214.69	260.12	2103.87	787.08	1971.63
Seed Yield/Plant	Cross 1	Mean	100.43	110.94	121.14	76.84	115.61	112.83
		Variance	452.45	615.64	165.97	1393.11	702.02	1199.84
	Cross 2	Mean	100.43	62.85	101.96	78.41	68.23	69.92
		Variance	452.45	364.13	687.20	1282.24	1086.99	883.56
	Cross 3	Mean	32.40	62.85	70.68	50.83	58.57	51.01
		Variance	136.50	364.13	359.69	871.34	593.13	696.41
chocolate spot	Cross 1	Mean	5.07	4.27	4.93	5.50	5.17	5.69
		Variance	0.21	0.21	0.21	2.69	1.45	1.76
	Cross 2	Mean	5.07	5.93	5.27	5.40	4.00	3.76
		Variance	0.21	0.35	0.44	1.63	0.90	1.18
	Cross 3	Mean	3.07	5.93	4.33	4.10	3.70	3.67
		Variance	0.21	0.35	0.38	1.67	1.04	1.13
Rust	Cross 1	Mean	6.20	6.07	5.00	5.73	5.47	6.25
		Variance	0.17	0.07	0.01	0.95	0.88	0.25
	Cross 2	Mean	6.20	6.43	7.13	6.68	6.00	4.87
		Variance	0.17	0.24	0.55	1.96	1.31	1.16
	Cross 3	Mean	4.80	6.43	5.73	5.83	4.23	4.70
		Variance	0.31	0.24	0.35	2.08	0.94	1.67

**Table 2:** Heterosis, inbreeding depression, and potence ratio of the three crosses for the studied traits.

Traits	Crosses	Heterosis		Inbreeding Depression	Potence Ratio
		BP	MP		
Plant Height	Cross 1	12.74 **	13.62 **	0.55	17.47
	Cross 2	38.82 **	26.40 **	20.37	−2.95
	Cross 3	−0.95	3.91 *	12.56	0.80
No. of Branches/plant	Cross 1	34.62 **	46.90 **	42.57 **	−5.14
	Cross 2	11.11 **	13.25 **	4.00	−6.88
	Cross 3	31.78 **	94.43 **	17.03 **	−1.99
No. of Pods/Plant	Cross 1	32.03 **	24.08 **	40.33 *	−4.00
	Cross 2	27.22 **	20.84 **	12.82	−4.15
	Cross 3	21.75 *	4.73 *	20.25	−0.34
No. of Seeds/Plant	Cross 1	40.97 **	24.29 **	31.84	−2.05
	Cross 2	50.17 **	50.93 **	24.99	100.7
	Cross 3	81.42 **	37.57 **	22.29	−1.55
Seed Yield/Plant	Cross 1	9.19 *	14.62 **	36.57	−2.94
	Cross 2	24.89 **	24.89 **	23.1	1.08
	Cross 3	12.46 **	48.41 **	28.08	−1.51
Chocolate spot	Cross 1	−2.76 **	5.57 **	−11.56 **	0.65
	Cross 2	−4.18 **	−4.18 **	−2.47	0.53
	Cross 3	41.04 **	−3.78 **	5.31 **	0.12
Rust	Cross 1	−19.35 **	−18.50 **	−14.60 **	−17.46
	Cross 2	12.91 **	12.91 **	6.31 **	−7.09
	Cross 3	19.38 **	2.05 **	−1.75	−0.14

* and ** significant at 0.05 and 0.01 levels of probability, respectively.

**Table 3:** Gene action (Gamble’s parameters) of the three crosses for the studied traits.

Traits	Crosses	Gamble’s Parameters					
		(m)	(a)	(d)	(aa)	(ad)	(dd)
Plant Height	Cross 1	120.33 **	5.30 **	51.90 **	37.40 **	4.47	−109.12 **
	Cross 2	118.65 **	6.05 **	−9.57	−40.69 **	16.59 **	140.54 **
	Cross 3	122.70 **	19.17 **	4.15	−1.13	12.55 **	62.23 **
No. of Branches/plant	Cross 1	4.02 **	1.29 **	5.18 **	2.94 **	1.73 **	1.57
	Cross 2	4.80 **	−0.18	1.75	1.16	−0.1	−2.69
	Cross 3	4.92 **	0.1	2.34	−0.55	1.55 **	−0.62
No. of Pods/Plant	Cross 1	29.51 **	−6.22 **	64.80 **	55.20 **	−3.82	−49.76 **
	Cross 2	41.56 **	−5.07	−11.08	−19.29 **	−3.09	46.58 **
	Cross 3	30.36 **	1.13	31.35 **	29.63 **	6.21	−31.86 **
No. of Seeds/Plant	Cross 1	107.88 **	−8.92	211.14 **	180.20 **	6.15	−220.71 **
	Cross 2	126.47 **	−6.75	−16.55	−73.44 **	−7.31	201.62 **
	Cross 3	95.68 **	17.2	70.24 **	36.61 **	38.84 **	−30.67 **
Seed Yield/Plant	Cross 1	76.84 **	2.78	164.98 **	149.52 **	8.04	−152.75 **
	Cross 2	78.41 **	−1.69	−17.02	−37.34	−20.48 **	128.26 **
	Cross 3	50.83 **	7.56	38.88 **	15.83	22.79 **	1.61
Chocolate spot	Cross 1	5.50 **	−0.52	−0.02	−0.28	−0.92 **	−2.24
	Cross 2	5.40 **	0.24	−6.32 **	−6.08 **	6.73 **	12.10 **
	Cross 3	4.10 **	0.03	−1.83 **	−1.67 **	1.46 **	4.60 **
Rust	Cross 1	5.73 **	−0.78 **	−0.62	0.52	−0.85 **	−1.69 *
	Cross 2	6.68 **	1.33 **	−4.57 **	−5.39 **	1.45 **	10.95 **
	Cross 3	5.83 **	−0.47	−5.33 **	−5.45 **	0.35	10.28 **

* and ** significant at 0.05 and 0.01 levels of probability, respectively.

**Table 4:** Estimates of heritability in broad (h^2^_b_) and narrow (h^2^_n_) senses plus genetic advance (GS) of the three crosses for the studied traits.

Traits	Crosses	Heritability		Genetic Advance	
		Broad Sense (h^2^_b_)	Narrow Sense (h^2^_n_)	GS	GS %
Plant Height	Cross 1	71.31	67.31	17.17	14.03
	Cross 2	77.79	51.83	12.11	10.21
	Cross 3	70.46	64.58	15.40	12.55
No. of Branches/plant	Cross 1	67.28	57.20	2.00	49.85
	Cross 2	70.92	63.47	2.57	53.52
	Cross 3	85.41	44.68	1.67	33.93
No. of Pods/Plant	Cross 1	80.25	74.05	23.7	80.3
	Cross 2	78.63	76.94	25.8	62.08
	Cross 3	86.05	65.54	22.37	73.68
No. of Seeds/Plant	Cross 1	88.94	75.94	83.67	77.55
	Cross 2	84.67	82.20	86.15	68.12
	Cross 3	90.10	68.87	65.08	68.02
Seed Yield/Plant	Cross 1	70.47	63.48	48.81	63.52
	Cross 2	57.28	46.32	34.17	43.58
	Cross 3	65.00	52.00	31.62	62.21
Chocolate spot	Cross 1	92.20	80.32	2.71	49.36
	Cross 2	77.91	72.39	1.90	35.26
	Cross 3	80.24	70.06	1.87	45.49
Rust	Cross 1	91.63	81.01	1.62	28.34
	Cross 2	80.74	73.98	2.13	31.94
	Cross 3	84.98	74.52	2.21	37.98

**Table 5:** Total bands (monomorphic and polymorphic), polymorphism percentage, and detected SCoT markers generated for resistance and susceptibility to foliar diseases using the seven SCoT markers across the selected faba bean lines.

Marker Name	Total Bands	MMB	PMB	% Polymorphism	Number of Bands Associated with	
					Resistance	Susceptibility
SCoT1	93	66	27	29	1 at 260 bp	---
SCoT2	77	66	11	14	---	1 at 327 bp
SCoT3	47	0	47	100	---	1 at 361 bp
SCoT4	54	0	54	100	1 at 207 bp	---
SCoT5	79	0	79	100	---	2 at 270 and 779 bp
SCoT6	56	0	56	100	---	1 at 392 bp
SCoT7	116	33	83	72	1 at 178 bp	---
Total	522	165	357	---	3	5
Average	75	24	51	74	---	---

Note: MMB: monomorphic band; PMB: polymorphic band.

**Table 6:** Indicators of genetic variability and information content of SCoT markers based on the analysis of the selected lines.

Index	Scored Band	H_0	PIC_0	E_0	H.av_0	MI_0	D_0	R_0
SCoT 1	93	0.416	0.330	2.818	0.0032	0.0089	0.505	0.485
SCoT 2	77	0.486	0.368	2.333	0.0037	0.0086	0.662	0.667
SCoT 3	47	0.459	0.353	1.424	0.0035	0.0049	0.875	1.455
SCoT 4	54	0.483	0.367	1.636	0.0037	0.0060	0.834	2.364
SCoT 5	79	0.480	0.365	2.394	0.0024	0.0058	0.842	3.152
SCoT 6	56	0.491	0.371	1.697	0.0050	0.0084	0.683	2.061
SCoT 7	116	0.418	0.330	3.515	0.0025	0.0089	0.507	2.545
Average		0.462	0.355	2.260	0.0034	0.0074	0.701	1.818

Note: H: expected heterozygosity; PIC: polymorphism information content; E: effective multiplex ratio; H.av: mean of heterozygosity; MI: marker index; D: discriminating power; R: resolving power.

**Table 7:** Pedigree, origin, and reaction to foliar diseases of four parental faba bean genotypes under study.

Genotype	Pedigree	Origin	Reaction to Foliar Diseases
Nubaria 5 (P_1_)	landraces of Hamam 10	* FCRI, ARC, Egypt	Resistant
Sakha 1 (P_2_)	Giza 716 × 620/283/85		Resistant
Giza 40 (P_3_)	Selection from Rebaya 40		Highly susceptible
Marina (P_4_)	*Vicia faba* L.	Hungary	Highly resistant

* FCRI: Field Crops Research Institute; ARC: Agricultural Research Center.

## Data Availability

The datasets used and/or analyzed during the current study are available from the corresponding author upon reasonable request.

## Supplementary Materials

The following supporting information can be downloaded at: https://www.mdpi.com/article/10.3390/plants12203645/s1, Table S1. Scaling test of the three crosses for the studied traits. Table S2. List of SCoT primers and their sequences. Table S3. Similarity coefficient (Dice measurement) of the selected genotypes based on SCoT banding profile. Figure S1. SCoT-PCR amplification using the selected primers.
