# Supplementary material for: The Potential Genetic Effect for Yield and Foliar Disease Resistance in Faba Bean (Vicia faba L.) Assessed via Morphological and SCoT Markers
Source: Plants (Basel). 2023 Oct 22;12(20):3645. doi: 10.3390/plants12203645 (PMC10610329; doi:10.3390/plants12203645)
Supplement: Supplementary file 1 [file plants-12-03645-s001.zip › plants-2582775-supplementary.pdf]

Tabel S1. Scaling test of the three crosses for the studied traits.

Traits	Crosses	Scaling test			
		A	B	C	D
Plant Height	Cross 1	40.33**	31.39**	34.32**	-18.70**
	Cross 2	-33.33**	-66.52**	-59.16	20.35
	Cross 3	-18.00**	-43.10**	-59.96**	0.57
No. of Branches/plant	Cross 1	-0.53	-3.98**	-7.45**	-1.47*
	Cross 2	0.67	0.86	0.37**	-0.58**
	Cross 3	2.13**	-0.97	1.71**	0.27
No. of Pods/Plant	Cross 1	-6.54	1.1	-60.64**	-27.60**
	Cross 2	-20.70**	-6.59	-7.99	9.65
	Cross 3	-2.83	5.07	-27.39**	-14.81**
No. of Seeds/Plant	Cross 1	26.4	14.11	-139.69**	-90.10**
	Cross 2	-71.40**	-56.77**	-54.73**	36.72**
	Cross 3	35.87**	-41.81**	-42.56**	-18.31**
Seed Yield/Plant	Cross 1	9.65	-6.42	-146.29**	-74.76**
	Cross 2	-65.93**	-24.98**	-53.57**	18.67**
	Cross 3	14.07	-31.51**	-33.27**	-7.91
Chocolate spot	Cross 1	0.34	2.18**	2.80**	0.14
	Cross 2	-2.33**	-3.68**	0.07	3.04**
	Cross 3	0.01	-2.93**	-1.26**	0.83**
Rust	Cross 1	-0.26	1.43**	0.65	-0.26
	Cross 2	-1.56**	-4.00**	-0.18	2.69**
	Cross 3	-3.70**	-1.13**	0.62	2.73**

Table S2. List of SCoT primers and their sequences.

Primer	Primer sequence (5`→ 3`)
SCoT-1	5'-ACGACATGGCGACCACGC-3'
SCoT-2	5'-ACCATGGCTACCACCGGC-3'
SCoT-3	5'-ACGACATGGCGACCCACA-3'
SCoT-4	5'-CAATGGCTACCACTAGCG-3'
SCoT-5	5'-CAATGGCTACCACTACAG-3'
SCoT-6	5'-ACAATGGCTACCACTGAC-3'
SCoT-7	5'-ACGACATGGCGACCCAC-3'

Figure S1. SCoT-PCR amplification using the selected primers

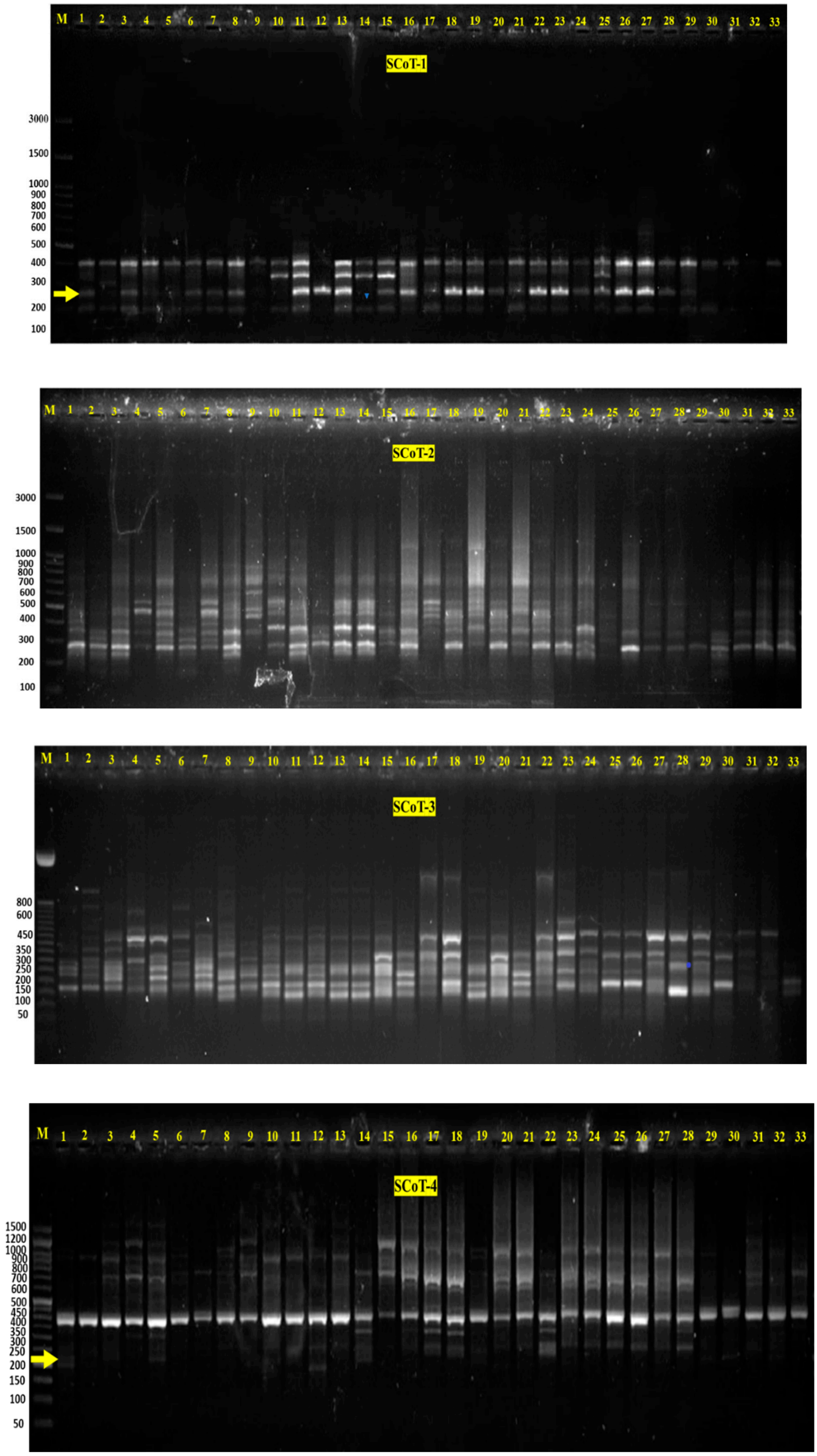

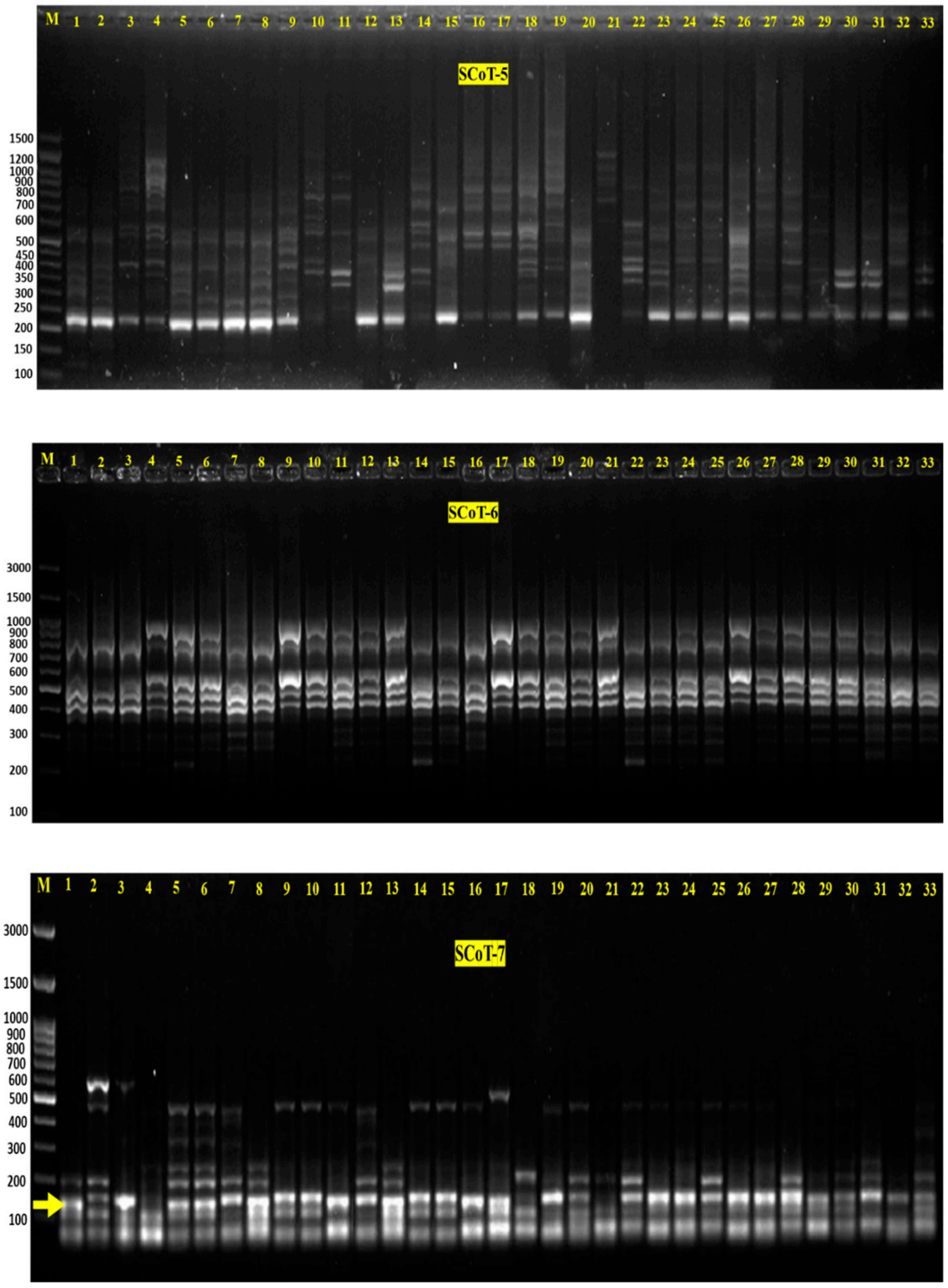
M 1 2 3 4 5 6 7 8 9 10 11 12 13 14 15 16 17 18 19 20 21 22 23 24 25 26 27 28 29 30 31 32 33
SCoT-5
1500
1200
1000
900
800
700
600
500
450
400
350
300
250
200
150
100
M 1 2 3 4 5 6 7 8 9 10 11 12 13 14 15 16 17 18 19 20 21 22 23 24 25 26 27 28 29 30 31 32 33
SCoT-6
3000
1500
1000
900
800
700
600
500
400
300
200
100
M 1 2 3 4 5 6 7 8 9 10 11 12 13 14 15 16 17 18 19 20 21 22 23 24 25 26 27 28 29 30 31 32 33
SCoT-7
3000
1500
1000
900
800
700
600
500
400
300
200
100

Table S3. Similarity coefficient (Dice measurement) of the selected genotypes based on SCoT banding profile.

**Marina**	**1.00**																																
**Giza 40**	0.79	**1.00**																															
**F 1**	0.73	0.74	**1.00**																														
**F2 (1)**	0.74	0.70	0.84	**1.00**																													
**F2 (2)**	0.73	0.74	0.78	0.84	**1.00**																												
**F2 (3)**	0.74	0.70	0.74	0.80	0.88	**1.00**																											
**F2 (4)**	0.67	0.74	0.63	0.74	0.88	0.84	**1.00**																										
**F2 (5)**	0.74	0.70	0.65	0.76	0.85	0.86	0.91	**1.00**																									
**F2 (6)**	0.64	0.60	0.55	0.53	0.42	0.47	0.50	0.52	**1.00**																								
**F2 (7)**	0.67	0.69	0.63	0.63	0.51	0.50	0.60	0.55	0.91	**1.00**																							
**F2 (8)**	0.69	0.76	0.65	0.65	0.59	0.53	0.56	0.58	0.83	0.85	**1.00**																						
**F2 (9)**	0.81	0.87	0.71	0.82	0.76	0.77	0.81	0.78	0.62	0.71	0.73	**1.00**																					
**F2 (10)**	0.82	0.93	0.73	0.78	0.77	0.78	0.82	0.79	0.65	0.73	0.74	0.95	**1.00**																				
**BC1 (1)**	0.81	0.71	0.59	0.65	0.65	0.71	0.69	0.71	0.75	0.69	0.71	0.73	0.74	**1.00**																			
**BC1 (2)**	0.77	0.67	0.56	0.61	0.61	0.67	0.65	0.67	0.70	0.64	0.67	0.69	0.71	0.96	**1.00**																		
**BC1 (3)**	0.77	0.67	0.61	0.67	0.61	0.67	0.65	0.67	0.78	0.72	0.74	0.69	0.71	0.96	0.92	**1.00**																	
**BC1 (4)**	0.75	0.65	0.76	0.76	0.70	0.59	0.63	0.58	0.50	0.62	0.57	0.67	0.63	0.64	0.67	0.67	**1.00**																
**BC1 (5)**	0.91	0.74	0.74	0.69	0.63	0.63	0.61	0.63	0.72	0.74	0.69	0.71	0.72	0.83	0.79	0.86	0.83	**1.00**															
**BC1 (6)**	0.76	0.77	0.76	0.77	0.86	0.77	0.81	0.83	0.55	0.65	0.73	0.79	0.80	0.67	0.63	0.69	0.67	0.71	**1.00**														
**BC1 (7)**	0.85	0.74	0.63	0.74	0.68	0.74	0.73	0.81	0.72	0.74	0.76	0.82	0.83	0.83	0.79	0.86	0.62	0.80	0.82	**1.00**													
**BC1 (8)**	0.84	0.61	0.61	0.67	0.61	0.67	0.65	0.67	0.78	0.72	0.67	0.69	0.71	0.89	0.85	0.92	0.67	0.86	0.69	0.86	**1.00**												
**BC1 (9)**	0.83	0.89	0.73	0.74	0.78	0.74	0.78	0.80	0.50	0.60	0.69	0.86	0.87	0.69	0.65	0.65	0.69	0.73	0.86	0.79	0.65	**1.00**											
**BC1 (10)**	0.80	0.92	0.75	0.70	0.70	0.65	0.69	0.71	0.59	0.69	0.77	0.78	0.84	0.71	0.67	0.73	0.71	0.81	0.83	0.81	0.67	0.91	**1.00**										
**BC2 (1)**	0.71	0.73	0.61	0.61	0.61	0.67	0.71	0.73	0.78	0.72	0.74	0.69	0.76	0.89	0.85	0.92	0.59	0.79	0.75	0.86	0.85	0.71	0.80	**1.00**									
**BC2 (2)**	0.78	0.74	0.68	0.68	0.73	0.74	0.72	0.74	0.64	0.67	0.69	0.70	0.77	0.75	0.71	0.77	0.63	0.79	0.86	0.85	0.77	0.78	0.80	0.84	**1.00**								
**BC2 (3)**	0.81	0.76	0.65	0.53	0.65	0.65	0.69	0.71	0.75	0.69	0.71	0.67	0.74	0.86	0.81	0.81	0.64	0.83	0.73	0.76	0.81	0.75	0.77	0.89	0.81	**1.00**							
**BC2 (4)**	0.67	0.69	0.57	0.56	0.57	0.63	0.67	0.69	0.82	0.75	0.77	0.65	0.73	0.85	0.80	0.88	0.54	0.74	0.71	0.81	0.80	0.67	0.76	0.96	0.80	0.85	**1.00**						
**BC2 (5)**	0.73	0.69	0.63	0.63	0.63	0.63	0.67	0.69	0.73	0.67	0.77	0.71	0.67	0.85	0.80	0.88	0.69	0.81	0.77	0.81	0.80	0.73	0.76	0.88	0.80	0.85	0.83	**1.00**					
**BC2 (6)**	0.69	0.82	0.65	0.59	0.65	0.59	0.63	0.65	0.58	0.69	0.79	0.67	0.74	0.64	0.67	0.67	0.64	0.69	0.79	0.76	0.59	0.81	0.90	0.74	0.75	0.71	0.77	0.69	**1.00**				
**BC2 (7)**	0.63	0.82	0.65	0.71	0.65	0.65	0.69	0.65	0.67	0.77	0.79	0.85	0.80	0.64	0.59	0.67	0.57	0.62	0.73	0.76	0.59	0.75	0.77	0.67	0.63	0.57	0.69	0.69	0.79	**1.00**			
**BC2 (8)**	0.73	0.80	0.74	0.80	0.63	0.63	0.67	0.69	0.72	0.81	0.83	0.88	0.83	0.69	0.64	0.71	0.69	0.73	0.76	0.80	0.64	0.79	0.81	0.71	0.67	0.62	0.74	0.74	0.76	0.90	**1.00**		
**BC2 (9)**	0.73	0.75	0.57	0.63	0.51	0.56	0.53	0.62	0.64	0.67	0.69	0.71	0.73	0.69	0.72	0.72	0.62	0.74	0.65	0.81	0.64	0.73	0.83	0.72	0.67	0.62	0.75	0.67	0.85	0.77	0.81	**1.00**	
**BC2 (10)**	0.73	0.75	0.57	0.56	0.57	0.56	0.53	0.62	0.64	0.67	0.77	0.65	0.67	0.69	0.72	0.72	0.62	0.74	0.71	0.81	0.64	0.73	0.83	0.72	0.73	0.69	0.75	0.75	0.92	0.77	0.74	0.92	**1.00**
